# Sequencing Analysis of *MUC6* and *MUC16* Gene Fragments in Patients with Oropharyngeal Squamous Cell Carcinoma Reveals Novel Mutations: A Preliminary Study

**DOI:** 10.3390/cimb45070356

**Published:** 2023-07-04

**Authors:** Jadwiga Gaździcka, Krzysztof Biernacki, Silvia Salatino, Karolina Gołąbek, Dorota Hudy, Agata Świętek, Katarzyna Miśkiewicz-Orczyk, Anna Koniewska, Maciej Misiołek, Joanna Katarzyna Strzelczyk

**Affiliations:** 1 Department of Medical and Molecular Biology, Faculty of Medical Sciences in Zabrze, Medical University of Silesia in Katowice, Jordana 19, 41-808 Zabrze, Poland; 2 Molecular Biology, Core Research Laboratories, Natural History Museum, London SW7 5BD, UK; 3 Silesia LabMed Research and Implementation Centre, Medical University of Silesia in Katowice, Jordana 19, 41-808 Zabrze, Poland; 4 Department of Otorhinolaryngology and Oncological Laryngology, Faculty of Medical Sciences in Zabrze, Medical University of Silesia in Katowice, C. Skłodowskiej 10, 41-800 Zabrze, Poland

**Keywords:** oropharyngeal squamous cell carcinoma (OPSCC), mutation, *MUC6*, *MUC16*

## Abstract

The growing incidence of oropharyngeal squamous cell carcinoma (OPSCC) calls for better understanding of the mutational landscape of such cases. Mucins (MUCs) are multifunctional glycoproteins expressed by the epithelial cells and may be associated with the epithelial tumour invasion and progression. The present study aimed at the analysis of the sequence of selected *MUC6* and *MUC16* gene fragments in the tumour, as well as the margin, samples obtained from 18 OPSCC patients. Possible associations between the detected mutations and the clinicopathological and demographic characteristics of the study group were analysed. Sanger sequencing and bioinformatic data analysis of the selected *MUC6* and *MUC16* cDNA fragments were performed. Our study found 13 and 3 mutations in *MUC6* and *MUC16*, respectively. In particular, one novelty variant found that the *MUC6* gene (chr11:1018257 A>T) was the most frequent across our cohort, in both the tumour and the margin samples, and was then classified as a high impact, stop-gain mutation. The current study found novel mutations in *MUC6* and *MUC16* providing new insight into the genetic alternation in mucin genes among the OPSCC patients. Further studies, including larger cohorts, are recommended to recognise the pattern in which the mutations affect oropharyngeal carcinogenesis.

## 1. Introduction

Mucins are multifunctional glycoproteins expressed by epithelial cells in a variety of tissues [1] and classified as membrane-bound or secreted mucins, the latter further divided into gel-forming and non-gel-forming ones [2]. Membrane-bound mucins are important in numerous biological processes, including molecular cell signalling [3]. Acting like receptors, they can conduct signals from the environment to the cell, therefore influencing proliferation, differentiation or apoptosis [4]. Some studies suggest an association between mutations in *MUC16* and the immune response and the cell cycle in cancer patients [5,6]. MUC16 has a role in the maintenance of the mucosa and acts as a barrier against external agents [7], such as bacterial adherence [8,9]. On the other hand, secreted mucins (e.g., MUC6) play a key role in the protection of the epithelium against infection agents, chemical injury or dehydration by forming a mucus layer [10]. MUC6 expresses O-glycans, which may take an important part in bacterial growth control [11]. Importantly, during carcinogenesis mucins may have an important role in cancer cell differentiation and metastasis [12].

In 2020, oropharyngeal cancer was diagnosed in 98,412 patients worldwide and caused the death of 48,143 people [13]. Most oropharyngeal cancers are squamous cell carcinomas (OPSCCs), arising from the mucosa epithelium of the oropharynx. As for all other types of head and neck squamous cell carcinomas (HNSCCs), multiple risk factors are known for OPSCCs: use of tobacco and alcohol, poor oral hygiene, ageing, environmental pollutants and viral infection, such as human papillomavirus (HPV) [14]. Favourably, HPV vaccines are effective against the virus, especially for genotypes HPV-16 and HPV-18, associated with OPSCC [15]. The median age for HPV-positive OPSCC is 53 and 58 years for HPV-negative [16]. OPSCC patients with HPV infection, whose number is increasing worldwide [17], have more favourable prognoses [18,19]. HPV-negative tumours located in the head and neck demonstrated different somatic mutations compared to HPV-positive HNSCC [20]. Sequencing analysis of HNSCC from different anatomic sites, with or without HPV, revealed a markedly diverse landscape of mutations [21]. Comparison of HPV-positive tonsillar OPSCC and HPV-negative oral squamous cell carcinoma (OSCC) revealed a mutation in *MUC12* shared by both types of cancers, and a higher number of mucin genes with mutations were noticed in HPV-positive OPSCC patients [22].

**Figure 1:**
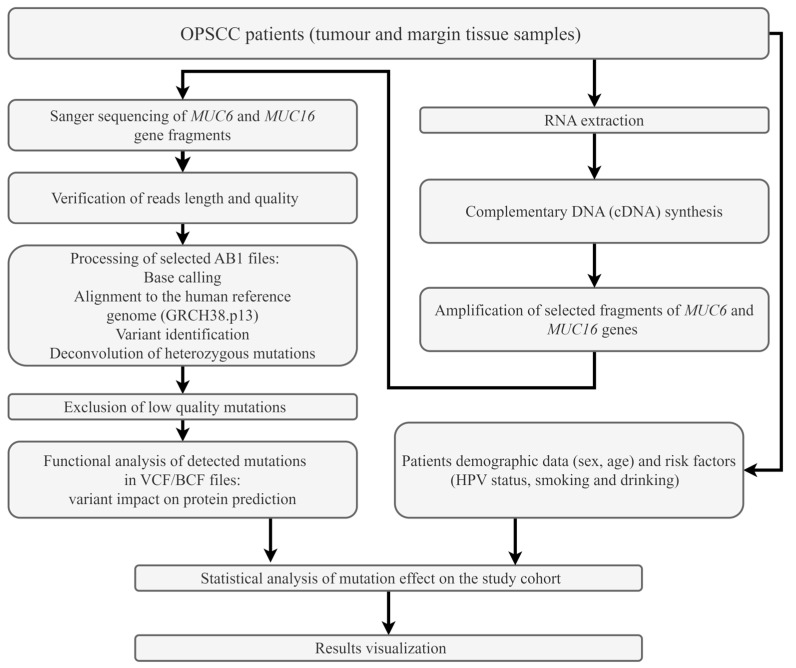
Flow chart of the study.

The present study aimed to analyse the sequence of some selected fragments of *MUC6* and *MUC16* genes in the tumour and the margin samples obtained from the OPSCC patients. Some possible associations between the detected mutations and the clinicopathological or demographic characteristics of the study group were also analysed. Moreover, the identified variants in *MUC6* and *MUC16* genes were evaluated in relation to their HPV status. Figure 1 presents the study flow chart.

## 2. Materials and Methods

### 2.1. Patients and Samples

The study population comprised 18 OPSCC patients recruited at the Department of Otorhinolaryngology and Oncological Laryngology in Zabrze, Medical University of Silesia in Katowice (Poland). The main inclusion criterion was the diagnosis of primary OPSCC, while the exclusion criteria included preoperative chemotherapy and radiotherapy. All of the cases derived from a Polish, white population. The key data (age, sex, medical history and use of tobacco and alcohol) were collected through an ad hoc questionnaire. The study was approved of by the Ethics Committee of the Medical University of Silesia (no. KNW/0022/KB1/49/16 and KNW/0022/KB1/49/II/16/17). Informed consent was obtained from all the patients enrolled in the study. This work was supported by a grant from the Medical University of Silesia (KNW-2-O07/N/9/N).

Two tissue samples were obtained from each of the OPSCC patients: a tumour sample and a histologically normal surgical margin sample. All samples were collected during the surgical resection. The anatomical location of the OPSCC samples comprised the palatine tonsils only. The tumour samples contained the histopathologically proven primary OPSCC cells. An R0 resection (microscopically margin negative resection) was performed in all the patients and the margin samples were histopathologically confirmed as free of cancerous cells and dysplasia. The tumour stage was categorised according to the International Union Against Cancer (UICC) classification of head and neck tumours (7th Edition) [23]. After resection, all specimens were immediately immersed in RNAlater^®^ (Sigma-Aldrich, Saint Louis, MO, USA) and frozen at −80 °C pending RNA extraction. Figure S1 shows the detailed procedures of our laboratory work.

### 2.2. MUC 6 and MUC16 Sequencing

#### 2.2.1. RNA Extraction

Homogenisation of each tumour and of the margin sample was the first step preceding RNA extraction and use of ceramic beads Lysing Matrix D (MP Biomedicals, Irvine, CA, USA) in FastPrep^®^-24 homogeniser (MP Biomedicals, Irvine, CA, USA). RNA extraction was performed with the use of an RNA isolation kit (BioVendor, Brno, Czech Republic) according to the manufacturer’s protocol. Estimation of the quality and quantity of the extracted RNA was performed on a NanoPhotometer^®^ Pearl spectrophotometer (IMPLEN, Munich, Germany).

#### 2.2.2. Complementary DNA (cDNA) Synthesis

A total of 5 ng RNA was reverse-transcribed into complementary DNA with the High Capacity cDNA Reverse Transcription Kit with RNase Inhibitor (Applied Biosystems™, Waltham, MA, USA) according to the manufacturer’s instructions. The reaction was run in a 20 μL volume. Briefly, 10 μL of the previously extracted RNA was added to 2 μL of 10× Buffer RT, 2 μL of 10× RT Random Primers, 0.8 μL of 25× dNTP mix (100 mM), 1 μL of MultiScribe™ Reverse Transcriptase, 1 μL of RNase inhibitor, and 3.2 μL of nuclease-free H_2_O. The thermal parameters of the reaction were as follows: 25 °C for 10 min, 37 °C for 120 min, 85 °C for 5 min and cooldown to 4 °C–∞. The reaction was performed in Mastercycler personal (Eppendorf AG, Hamburg, Germany).

#### 2.2.3. Amplification of Selected Fragments of MUC6 and MUC16 Genes

Both sets of primers were designed using Primer-BLAST 3 (NCBI, Bethesda, MD, USA) to target all mRNA sequences of *MUC6* and *MUC16* genes. The primers were synthesised by Genomed (Genomed Joined-Stock Company, Warsaw, Poland), and their sequences are listed in Table 1. The first criterion for the amplified product selection was the presence of known variants in the central part of the amplified sequence. The secondary condition was the presence of the amplified fragment throughout all the known transcription variants of the gene.

The PCR reaction was run in a 20 μL volume. The amplification reaction was accomplished with Platinum™ II Hot-Start PCR Master Mix (2X) (Invitrogen, Waltham, MA, USA), according to the manufacturer’s protocol. Briefly, 10 μL of Platinum™ II Hot-Start PCR Master Mix was mixed with 0.4 μL 10 μM forward primer, 0.4 μL 10 μM reverse primer, 1 μL cDNA and 8.2 μL nuclease-free water. The PCR conditions were as follows: denaturation at 94 °C for 2 min, and 35 cycles of 94 °C for 15 s, 60 °C for 15 s and 68 °C for 15 s.

#### 2.2.4. Purification of the Selected MUC6 and MUC16 Gene Amplificated Fragments

Purification of the amplification products was performed with ExoSAP-IT™ Express PCR Product Cleanup Reagent (Applied Biosystems™, Waltham, MA, USA), according to the manufacturer’s instructions. Briefly, 5 μL of each PCR product was mixed with 2 μL ExoSAP-IT™ Express PCR Product Cleanup Reagent, vortex and incubated at 37 °C for 4 min and 80 °C for 1 min.

#### 2.2.5. Cycle Sequencing Reaction

The cycle sequencing reaction was performed with BigDye™ Terminator v3.1 Cycle Sequencing Kit (Applied Biosystems™, Waltham, MA, USA), according to the manufacturer’s protocol. The 3 μL of the purified PCR product was added to the reaction mixture containing 2 μL BigDye™ Terminator v3.1 Ready Reaction Mix, 1 μL of 5 × Sequencing Buffer, 3 μL deionised water and 1 μL forward/reverse primer (3.2 μM). The sequencing reaction was run in the following conditions: 96 °C for 1 min, and 25 cycles of 96 °C for 10 s, 50 °C for 5 s and 60 °C for 4 min. The reaction was run in QuantStudio 5 Real-Time PCR system (Applied Biosystems™, Waltham, MA, USA).

#### 2.2.6. Purification of the Templates and Capillary Electrophoresis

BigDye XTerminator^TM^ Purification Kit (Applied Biosystems™, Waltham, MA, USA) was used to purify the DNA sequencing reaction and prepared according to manufacturer’s protocol. Briefly, 55 μL of SAM/BigDye XTerminator^TM^ bead working solution was added to each sample on the sequencing plate and vortexed for 30 min at 2000 rpm (IKA MS3 Digital, IKA Werke GmbH&Co. KG, Staufen, Germany). The last step was to centrifuge the plate at 1000× *g* for 2 min. The supernatant of each sample was analysed in a 3130 Genetic Analyzer (Applied Biosystems™, Waltham, MA, USA) using 3130 POP-7^TM^ Performance-Optimized Polymer (Thermo Fisher Scientific, Waltham, MA, USA). The results were saved in the 3130 Genetic Analyzer software (Applied Biosystems™, Waltham, MA, USA).

### 2.3. Bioinformatic Analysis

Analysis of the Sanger sequencing data (AB1 files), including base calling, alignment to the human reference sequence (genome assembly GRCh38.p13), variant identification and deconvolution of heterozygous mutations, were performed using Tracy [24]. BCFtools (version 1.8) was used to handle VCF/BCF files, whereas all downstream functional annotations and predictions, including the variant impact on protein, SIFT (version sift5.2.2) [25] and PolyPhen-2 (version 2.2.2) scores [26], were determined using Variant Effect Predictor (VEP v103) [27]. All the acquired traces were manually inspected before the analysis to reject those showing very short sequences (fewer than 200 base pairs) using Chromatogram Explorer Lite (version v5.0.2, Heracle BioSoft, Pitesti, Romania).

### 2.4. Statistical Analysis and Data Visualisation

A comparison between the tumour and of the corresponding margin samples was performed using the Kruskal–Wallis test for age, TNM staging and histological grading, and *MUC6* and *MUC16* mutation occurrences. Fisher’s exact test was used to compare the tumour and the margin samples in regard of sex, smoking, alcohol drinking, *MUC6* and *MUC16* mutation occurrences and HPV status. Lollipop plots of mutation locations in *MUC6* and *MUC16* cDNA fragments were generated using the TrackViewer R library [28]. Hierarchical clustering using Euclidean distance calculation and its visualisation as heatmaps with dendrograms was performed using the gplots R library [29]. Analyses were performed with the use of R 4.2.2 in RStudio version 2022.12.0 build 353 (PBC, Boston, MA, USA) using the stats R library [30].

## 3. Results

### 3.1. Study Group

The study group included 18 patients, 4 of whom (22.22%) were women and 14 (77.78%) men, with a mean age of 62.83 ± 8.13. Seven of the patients (38.89%) were smokers. Ten cases (55.56%) admitted consuming alcohol; five (27.78%) consumed alcohol occasionally, and five (27.78%) drank alcohol on a regular basis. Our previous study delivered information about the HPV status of the cohort [31], where there were 12 (66.67%) HPV-positive patients, 5 (27.28%) HPV-negative individuals with only 1 case (5.56%) on which no information was available. Clinical data of the patients are presented in Table 2.

### 3.2. Sequencing Analysis

The analysis excluded samples with poor Sanger data quality and the final dataset was composed of 30 samples: 16 (53.33%) with OPSCC tumour and 14 (46.67%) margin samples. We obtained 25 sufficient quality traces of the *MUC6* fragment and 22 of the *MUC16* fragment. The list of used and rejected (based on quality) traces of the sequencing samples is shown in Table S1, along with the tumour and the margin sample ID for each patient.

A total of 30 different mutations were found across the cohort in the *MUC6* cDNA fragment. Similarly, 15 mutations were detected in the *MUC16* cDNA fragment. After evaluation and removal of the low-quality variants, the changes narrowed down to 13 mutations in the *MUC6* fragment and 3 variants the *MUC16* fragment. The example electropherograms of Sanger sequencing traces for *MUC6* and *MUC16* fragments with mutations are presented in Figure S2.

#### 3.2.1. Sequencing Analysis of MUC6

Analysis of the sequencing results for *MUC6* reported a total of 30 variants, 17 of which had low quality (hence, not used for further analyses) and the remainder 13 with good quality. Most single nucleotide variations (SNVs) were missense variants with transversion C>T. All mutations found in the *MUC6* fragment are presented in Table 3, while more detailed information is gathered in Table S2.

We observed similar occurrences of mutations in the tumour and the margin samples (*p* = 0.568). The highest number of variants in a single tumour sample was three, while in a single margin sample it was two. Moreover, we reported that all of the detected variants in *MUC6* corresponded to amino acidic changes. The mutation occurrences are summarised in Table 4.

**Figure 2:**
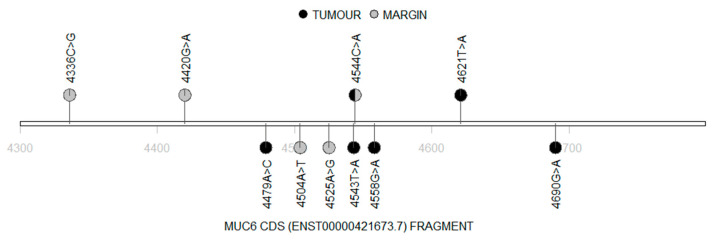
Lollipop diagram demonstrating the nucleotide changes in the mutated *MUC6* cDNA (black: tumour; grey: margin).

**Figure 3:**
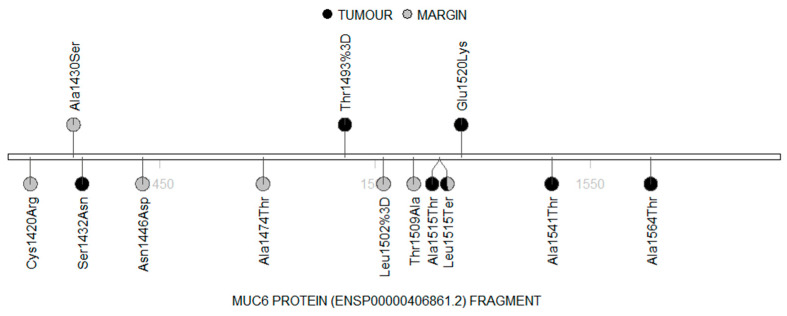
Lollipop diagram demonstrating the amino acid changes in the MUC6 protein fragment (black: tumour; grey: margin).

Interestingly, we found that most of the patients (16 cases) hosted the same stop-gained mutation in the *MUC6* gene (chr11:1018257 A>T; ENST00000421673.7:c.4544C>A), which was present neither in The Genome Aggregation Database (gnomAD) [32] nor in The Single Nucleotide Polymorphism database (dbSNP) [33]. This change was found in ten tumour samples and ten margin samples. Moreover, four patients had this mutation in their tumour and margin samples simultaneously. Furthermore, two of the known variants were found within the *MUC6* fragment: one had a moderate impact (COSV70139063) and the second had a low impact (COSV70138702) (Table S2). Distribution of the detected mutations in the *MUC6* cDNA fragment is presented in Figure 2, while the distribution of the single amino acid variants (SAVs), along with the MUC6 protein sequence, is presented in Figure 3.

We used the SIFT and PolyPhen prediction tools to analyse the effect of amino acid changes upon the structure and function of mucin. Using the PolyPhen score, one of the detected variants (c.4258G>C) was flagged as a probably damaging MUC6 protein structure. The values of SIFT and PolyPhen score analysis were 0.06 and 0.921, respectively. Moreover, SIFT predicted five variants as “tolerated” (two tolerated and three tolerated with low confidence) and three with “deleterious effect” (one deleterious and two deleterious with low confidence). PolyPhen predicted eight variants as “benign”. Detailed information on the SIFT and PolyPhen analyses are presented in Table S2.

#### 3.2.2. Sequencing Analysis of MUC16

Analysis of the sequencing results for *MUC16* reported a total of 15 variants, 12 of which had low quality (hence, not used for further analyses) and the remaining 3 had good quality. Two of the three SNVs were missense variants and one was a synonymous variant. Found in 12 patients, the most common variant type was a missense one located at position 8,964,498 of chromosome 19 (ENST00000397910.8:c.12272T>A). Moreover, three patients hosted this variant in the tumour and the margin samples simultaneously. Our analysis reported that no known variants occurred in the selected fragment of *MUC16*. All mutations identified in *MUC16* are shown in Table 5, while detailed information is gathered in Table S3.

**Figure 4:**
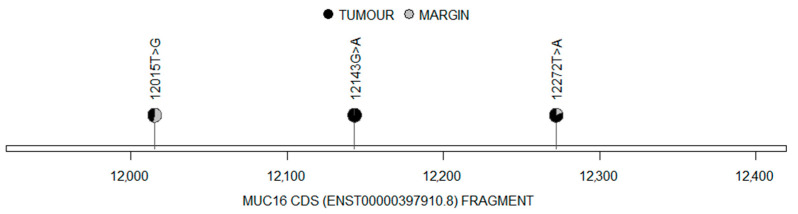
Lollipop diagram demonstrating the nucleotide changes in the mutated *MUC16* cDNA (black: tumour; grey: margin).

**Figure 5:**
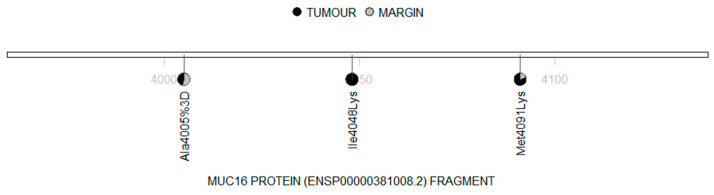
Lollipop diagram demonstrating the amino acid changes in the MUC16 protein fragment (black: tumour; grey: margin).

All the detected mutations corresponded to protein alterations and no significant difference in mutation occurrence was observed between the tumour samples and the margin samples (*p* = 0.946). Distribution of the detected mutations in the *MUC16* cDNA fragment is shown in Figure 4, while the distribution of the mutations found in the MUC16 protein sequence is shown in Figure 5.

Additionally, the first detected variant (ENST00000397910.8:c.12272T>A) was predicted with SIFT tool as “tolerated” (tolerated with low confidence, value 0.09) being a replacement of isoleucine (Ile) with lysine (Lys). The second detected variant (ENST00000397910.8:c.12143G>A) was predicted as “deleterious effect” (deleterious low confidence, the value of the SIFT score analysis was 0.0), as a consequence of the change from methionine (Met) to lysine (Lys). Analysis with the PolyPhen tool pointed to both variants as “benign” (0.013 and 0.028, respectively).

### 3.3. HPV Presence and MUC6 and MUC16 Mutations

We analysed the potential association between the HPV status and mutations of the mucin genes. No significant differences were found between the occurrences of mutations in *MUC6* or *MUC16* in HPV-positive tumour, as compared to HPV-negative tumour samples. Similarly, no significant results were observed in the margin samples.

### 3.4. Impact of the Common Occurrences of MUC6 and MUC16 Mutations on Clinicopathological and Demographic Characteristics of the Study Groups

There were no significant differences in mutation occurrences of *MUC6* between the tumour and the margin samples. No significant correlation was found between the parameters (age, TNM staging and histological grading) and the *MUC6* mutation frequency observed throughout the study group. In addition, no significant differences were observed between the mutation occurrence depending on demographic parameters (such as sex, smoking status, alcohol drinking status: casual, regular or general) in tumour or margin samples. Similarly, no significant differences were observed between mutation occurrences of *MUC16* and the clinicopathological or demographic characteristics for tumour or margin samples.

### 3.5. Mutation Clustering

**Figure 6:**
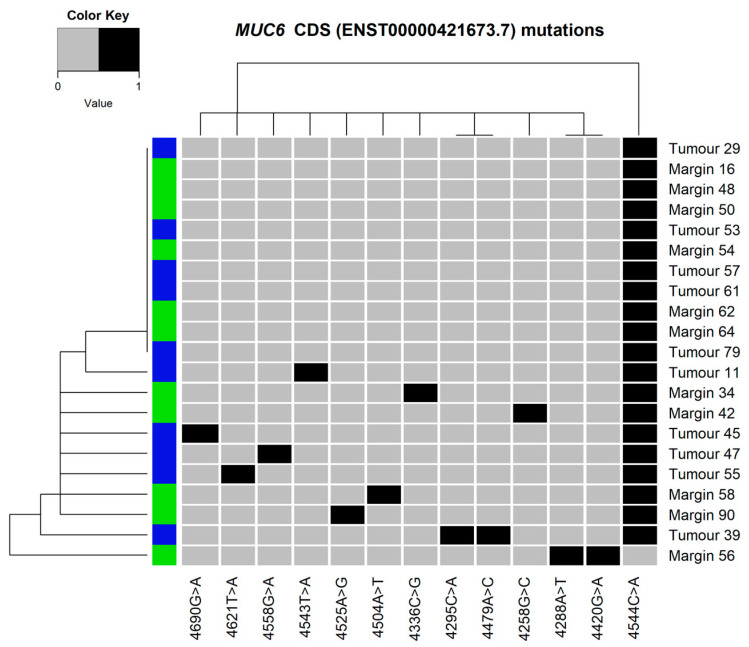
Heatmap and dendrograms presenting the mutations found in the selected fragment of *MUC6* coding sequence (CDS ENST00000421673.7). Tissue type shown as blue (tumour) or green (margin).

**Figure 7:**
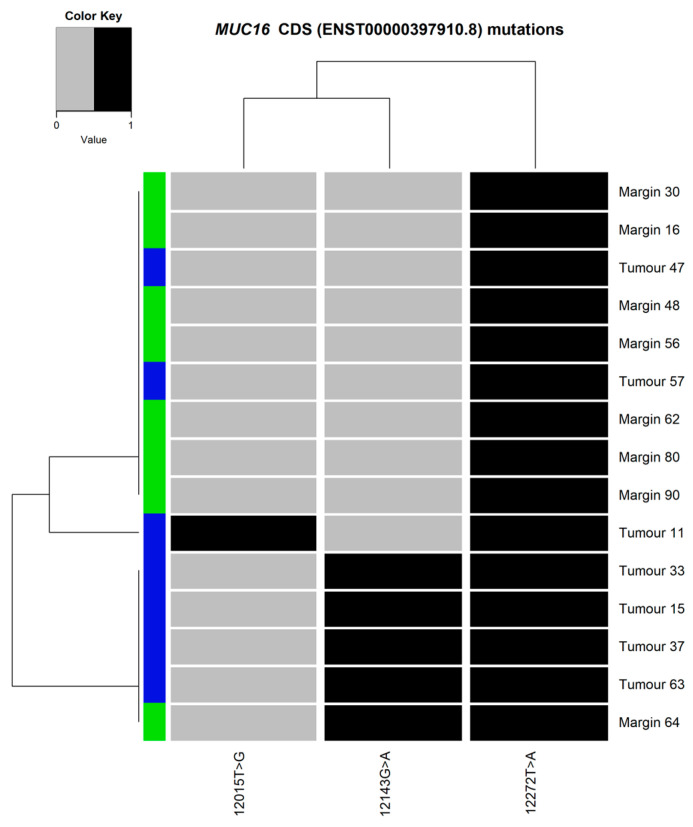
Heatmap and dendrograms presenting the mutations found in the selected fragment of *MUC16* coding sequence (CDS ENST00000397910.8). The tissue type illustrated as blue (tumour) or green (margin).

**Figure 8:**
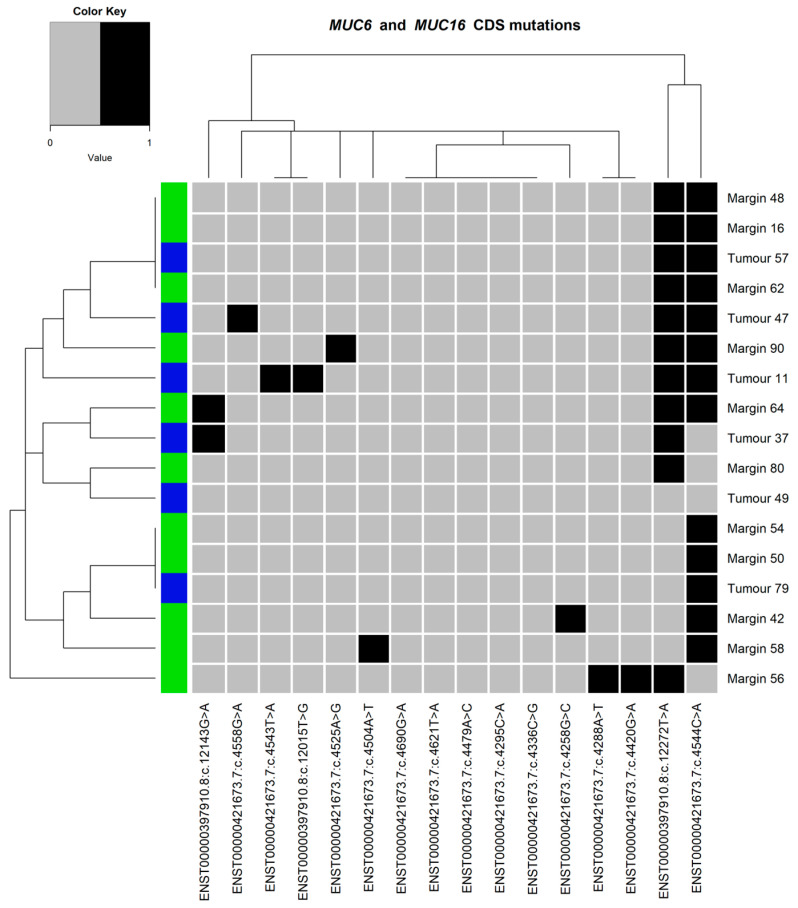
Heatmap and dendrograms presenting the *MUC6* coding sequence (CDS ENST00000421673.7) and *MUC16* coding sequence (CDS ENST00000397910.8) mutations. The tissue type shown as blue (tumour) or green (margin).

The heatmaps and dendrograms presenting the mutations found in *MUC6* and *MUC16* are shown in Figure 6 and Figure 7, respectively. Similarly, analyses for both genes, *MUC6* and *MUC16*, are presented in Figure 8. The hierarchical cluster analysis performed for the identified variants revealed no significant similarities nor mutation patterns in the *MUC6* coding sequence (Figure 6) or both *MUC6* and *MUC16* (Figure 8) corresponding to the tumour or margin groups. The mutational patterns did not correspond to any of the clustering data available in this study (TNM staging and histological grading, sex, alcohol drinking or smoking).

*MUC16* cDNA (Figure 7) mutations patterns showed that the presence of ENST00000397910.8:c.12272T>A and ENST00000397910.8:c.12143G>A together persisted frequently in the tumour samples (four out of seven samples) but not in the margin tissue samples (one out of eight samples). In one patient, both mutations were found in the tumour and the margin samples (sample IDs 63 and 64). Another patient had both mutations in the tumour sample but not in the corresponding margin sample (samples IDs 15 and 16).

## 4. Discussion

The growing number of OPSCC cases worldwide requires continuous research to better understand the molecular pathogenesis and to find potential targets for the effective cancer therapy. Mucins are glycosylated proteins known to facilitate tumour invasion and metastasis [4]. In the present study, we conducted a sequencing analysis of the selected fragments of *MUC6* and *MUC16* in 30 samples collected from the OPSCC patients. We detected 13 and 3 somatic mutations in *MUC6* and *MUC16*, respectively. The changes were mostly missense mutations. The selection of genes for sequencing analysis based on literature, which showed that mucin genes often mutated in HNSCC patients. However, the potential impact of somatic mutations in these genes on OPSCC is still poorly understood. Kannan et al. [34] detected numerous missense mutations in mucin genes, including *MUC6* and *MUC16*, in tonsil samples obtained from HPV-16 positive patients with OPSCC. In addition, Ährlund-Richter et al. [35] showed that *MUC6* and *MUC16* were mutated in over 30% of primary HPV-positive tonsillar squamous cell carcinoma (TSCC) and base of tongue squamous cell carcinoma (BOTSCC) with and without recurrence. In addition, Haft et al. [36] found mutations in other mucin genes (*MUC4* and *MUC5B*) in HPV-positive OPSCC patients from The Cancer Genome Atlas database (TCGA). It has been found that mutated *MUC6* and *MUC16* were involved in pathways associated with the extracellular matrix and carbohydrates in patients with TSCC and BOTSCC [35]. It was shown that nine out of nineteen mucin genes (including *MUC6* and *MUC16*) were frequently mutated in various cancer types, including HNSCC [37]. Overexpression of mucins is associated with proliferation, migration and invasion in various epithelial cancers [38]. Moreover, the frequency of mutations in mucins has been suggested to have an impact on cancer patients’ survival [39], indicating that mucins may be an important target of further analyses.

Our sequencing analysis of the *MUC6* fragment revealed some known mutations, confirmed also in COSMIC (Catalogue of Somatic Mutations in Cancer, version 96) [40] and detected in different cancers. The somatic variant COSV70128702 has been previously reported in colorectal cancer [41], while COSV70139063 was found in patients with basal cell carcinoma [42]. Interestingly, we identified a novel stop-gained mutation in the *MUC6* gene (ENST00000421673.7:c.4544C>A), which is not present in the gnomAD database [32] or in dbSNP [33] and classified as one with a potentially high impact on the downstream protein product. Detected in 16 patients, this somatic variant was the most common in our study. Moreover, it was found in both: the tumour and the margin samples. It suggests that it could be a population-specific polymorphism. However, further studies are needed to confirm or reject this hypothesis. As predicted by PolyPhen, another variant (ENST00000421673.7:c.4258G>C) may impact MUC6 protein structure in probably the damaging ways. Further analyses are required to investigate the potential influence of such changes on protein function or gene expression, especially in the case of the identified stop-gain mutation in *MUC6*. Mutations in *MUC6* were analysed in different cancers. Rokutan et al. [43] observed *MUC6* mutating in 20% of gastric dysplasia/intraepithelial neoplasia samples. Moreover, *MUC6* mutations were found in Epstein–Barr virus (EBV)-associated lymphoepithelioma-like cholangiocarcinoma [44], and directly associated with thyroid cancer [45]. On the other hand, the mutation status of *MUC6* and three other genes (*ATR*, *ERBB3* and *KDR*) was obtained as a marker for the recurrence of non-small-cell lung cancer (NSCLC) patients [46]. In addition, Shi et al. [47] identified that mutation in *MUC6* in Chinese patients with hepatocellular carcinoma, was associated with early recurrence. Interestingly, patients with stomach adenocarcinoma and mutated *MUC6* had better overall survival prognose, as compared to patients with wild-type *MUC6* [37]. Our study reveals no associations between *MUC6* mutations and the clinicopathological or demographic parameters of the OPSCC patients. However, the study group size was limited, therefore we plan to conduct further studies and statistical analysis with a larger study group.

Our study identified a few mutations in the fragment of *MUC16* cDNA. We found that ENST00000397910.8:c.12272T>A was the most common missense variant detected in *MUC16* in the tumour and the margin samples of OPSCC patients, which suggests that it could also be a population-specific polymorphism. Another variant ENST00000397910.8:c.12143G>A was found mostly in the tumour samples. Interestingly, we observed that these two mutations also occurred in one margin sample. The margin samples were verified as histopathologically free of cancerous cells. However, this tissue might still contain a minimal residual number of cancer cells, which are not detected by routine diagnostic methods [48]. Further investigations are required to verify this hypothesis. However, Kloss-Brandstätter et al. [49] detected mutation in mtDNA in OSCC patients and showed that some mutations were observed in tumour and resection margin samples. Our analysis revealed no association between mutation frequency in *MUC16* and the clinicopathological parameters, such as T or histological grading. Similarly, no significant associations were observed between the detected SNVs of *MUC16* and the demographic parameters. Interestingly, some studies observed that mutated *MUC16* influence on survival or prognosis in various cancers; Liu et al. [5] found that *MUC16* mutations were associated with better overall survival in patients with hepatocellular carcinoma, whereas Wang et al. [50] found that *MUC16* mutations were associated with overall survival in patients with melanoma. In addition, mutated *MUC16* was shown to improve the survival prognoses in patients with skin cutaneous melanoma [37], as well as in patients with gastric cancer [6,51]. Therefore, we design further studies assuming the use of an expanded cohort to determine whether such mutations could affect the OPSCC patient survival.

It has been observed that HPV-positive HNSCC had a mutational landscape different than HPV-negative tumours [22,52]. In our study, the frequencies of mutations in *MUC6* or *MUC16* did not correlate with HPV presence, although that might probably be due to the small size of our cohort. However, this could also be due to the location of the samples, all collected from the tonsils. Similarly to our results, Plath et al. [53] observed no significant differences in the total mutation counts and HPV-16 status in HNSCC patients, including the oropharynx site. Nichols et al. [22] found more mutations in an HPV-positive OPSCC patient than in an HPV-negative oral squamous cell carcinoma patient. In another study, HPV-positive patients with TSCC and BOTSCC hosted significantly fewer mutations than patients without HPV and the distribution of mutations was also different in the various genes analysed [51]. On the other hand, Gillison et al. [54] found no different significant mutation rates between HPV-positive HNSCC (mostly located in oropharyngeal) and HPV-negative HNSCC (mostly oral cavity). However, they reported that the most significantly mutated genes were different among patients with and without HPV, and suggested that the viral-host interaction may have an influence on genome characteristic of HPV-positive patients with oral and oropharyngeal squamous cell carcinoma [54].

The main limitations of the present study were small sample size and only one cDNA fragment of the selected mucins investigated. Further studies on a larger group are necessary to confirm the obtained results and to analyse the impact of the detected mutations on 5-year survival and recurrence.

## 5. Conclusions

The present study investigated multiple mutations identified from the selected fragments of *MUC6* and *MUC16* in the tumour and the surgical margin samples collected from the tonsillar tissue of OPSCC patients. We found that the most common stop-gain variant across the cohort was a novel mutation, located in the exonic region of the *MUC6* gene.

Our findings indicate that some of the investigated variants have a potentially deleterious impact on the structure of their protein products and will therefore require further investigations. The current study provides a new insight into the genetic alternation in mucin genes among the OPSCC patients. However, more extensive studies are needed to understand how such mutations may affect oropharyngeal carcinogenesis and survival and to verify whether they are polymorphisms specific to the Polish population.

## Figures and Tables

**Table 1:** Primer sequences for the *MUC6* and *MUC16* transcripts fragments.

Gene	Forward Primer 5′—3′	Reverse Primer 5′—3′
*MUC6* fragment (606 bp)	GAAGGATGTTGCCGTCATGG	ACTGAATACACAACGCCCCA
*MUC16* fragment (653 bp)	ACAGGCTGGGTCACAAGTTC	GGCGAGGTTGTAGCATGGAT

**Table 2:** Detailed clinical data of the patients.

Parameter	n	%
T1	3	16.67
T2	6	33.33
T3	9	50.00
N0	7	38.89
N1	1	5.56
N2	9	50.00
N3	1	5.56
G1	4	22.22
G2	8	44.44
G3	6	33.33

**Table 3:** Characteristics of the mutations identified in the *MUC6* gene.

Type Tissue (ID Sample)	POS	REF	ALT	HGVSc	HGVSp	Consequence
tumour (11, 29, 39, 45, 47, 53, 55, 57, 61, 79); margin (16, 34, 42, 48, 50, 54, 58, 62, 64, 90)	1018257	A	T	ENST00000421673.7:c.4544C>A	ENSP00000406861.2:p.Leu1515Ter	stop gained
margin (11)	1018258	C	T	ENST00000421673.7:c.4543T>A	ENSP00000406861.2:p.Ala1515Thr	missense variant
margin (34)	1018465	T	C	ENST00000421673.7:c.4336C>G	ENSP00000406861.2:p.Asn1446Asp	missense variant
tumour (39)	1018322	A	G	ENST00000421673.7:c.4479A>C	ENSP00000406861.2:p.Thr1493%3D	synonymous variant
tumour (39)	1018506	C	T,A	ENST00000421673.7:c.4295C>A	ENSP00000406861.2:p.Ser1432Asn	missense variant
margin (42)	1018543	A	G	ENST00000421673.7:c.4258G>C	ENSP00000406861.2:p.Cys1420Arg	missense variant
tumour (45)	1018111	C	T,A	ENST00000421673.7:c.4690G>A	ENSP00000406861.2:p.Ala1564Thr	missense variant
tumour (47)	1018243	C	T	ENST00000421673.7:c.4558G>A	ENSP00000406861.2:p.Glu1520Lys	missense variant
tumour (55)	1018180	C	T	ENST00000421673.7:c.4621T>A	ENSP00000406861.2:p.Ala1541Thr	missense variant
margin (56)	1018381	C	T	ENST00000421673.7:c.4420G>A	ENSP00000406861.2:p.Ala1474Thr	missense variant
margin (56)	1018513	C	A,T	ENST00000421673.7:c.4288A>T	ENSP00000406861.2:p.Ala1430Ser	missense variant
margin (58)	1018297	G	A	ENST00000421673.7:c.4504A>T	ENSP00000406861.2:p.Leu1502%3D	synonymous variant
margin (90)	1018276	T	C,A	ENST00000421673.7:c.4525A>G	ENSP00000406861.2:p.Thr1509Ala	missense variant

ID—sample identifier; POS—positions in chromosome; REF—reference nucleotide; ALT—detected nucleotide.

**Table 4:** The occurrences of mutations detected in the fragments of *MUC6* and *MUC16*.

Fragment	Sample	n (%)	Mean	SD	Minimum	Maximum	*p*-Value
*MUC6* cDNA	Tumour	10 (62.5%)	1.00	0.97	0	3	0.568
	Margin	11 (78.57%)	1.14	0.77	0	2	0.568
*MUC16* cDNA	Tumour	7 (43.75%)	0.75	0.93	0	2	0.946
	Margin	8 (57.14%)	0.64	0.63	0	2	0.946

**Table 5:** Characteristics of the mutations identified in the *MUC16* gene.

Type Tissue (ID Sample)	POS	REF	ALT	HGVSc	HGVSp	Consequence
tumour (11, 15, 33, 37, 47, 57, 63); margin (16, 30, 48, 56, 62, 64, 80, 90)	8964498	A	T	ENST00000397910.8:c.12272T>A	ENSP00000381008.2:p.Met4091Lys	missense variant
tumour (11); margin (16)	8964755	A	C/G,T	ENST00000397910.8:c.12015T>G	ENSP00000381008.2:p.Ala4005%3D	synonymous variant
tumour (15, 33, 37, 63); margin (64)	8964627	A	T	ENST00000397910.8:c.12143G>A	ENSP00000381008.2:p.Ile4048Lys	missense variant

ID—sample identifier; POS—positions in chromosome; REF—reference nucleotide; ALT—detected nucleotide.

## Data Availability

The data used to support the findings of this study are available from the corresponding author upon request.

## Supplementary Materials

The following supporting information can be downloaded at: https://www.mdpi.com/article/10.3390/cimb45070356/s1, Figure S1: The workflow diagram of wet-laboratory steps (samples preparation and sequencing reaction); Figure S2: Electropherograms of Sanger sequencing traces for *MUC6* ENST00000421673.7 and *MUC16* ENST00000397910.8; Table S1: Accepted and rejected sequencing results; Table S2: Detailed information of investigated *MUC6* (ENSG00000184956) mutations; Table S3: Detailed information of investigated *MUC16* (ENSG00000181143) mutations.
