# Supplementary material for: Sequencing Analysis of MUC6 and MUC16 Gene Fragments in Patients with Oropharyngeal Squamous Cell Carcinoma Reveals Novel Mutations: A Preliminary Study
Source: Curr Issues Mol Biol. 2023 Jul 4;45(7):5645–61. doi: 10.3390/cimb45070356 (PMC10377947; doi:10.3390/cimb45070356)
Supplement: Supplementary file 1 [file cimb-45-00356-s001.zip › cimb-2466412-supplementary.pdf]

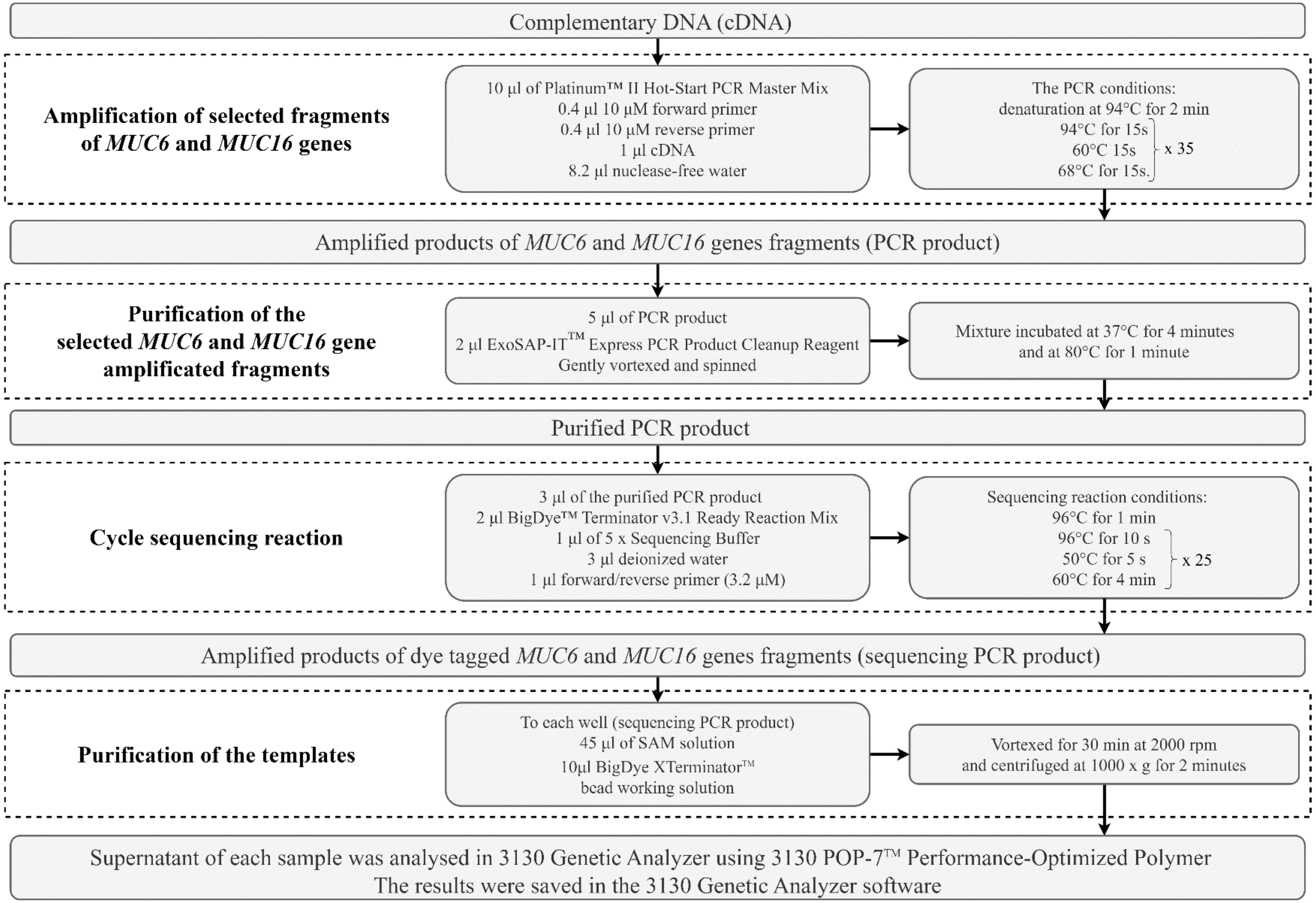

Figure S1. The workflow diagram of wet-laboratory steps (samples preparation and sequencing reaction).

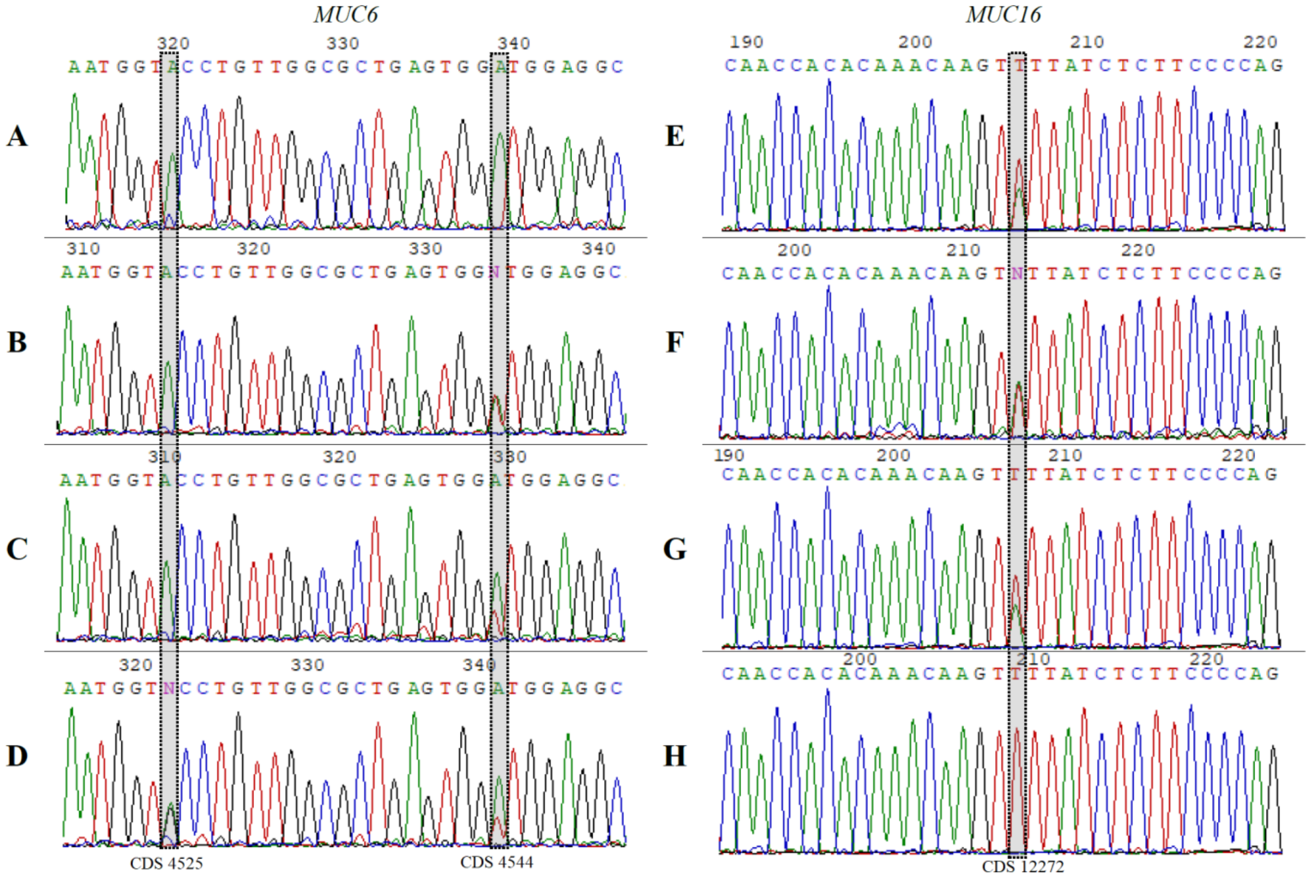

Figure S2. Electropherograms of Sanger sequencing traces for *MUC6* ENST00000421673.7 (A, B, C, D) and *MUC16* ENST00000397910.8 (E, F, G, H). (A) The absence of mutation A>G in CDS at position 4525 and the presence of a mutation C>A in CDS at position 4544 were found in the *MUC6* fragment from tumour sample ID 39. (B) The absence of mutation A>G in CDS at position 4525 and the presence of a mutation C>A in CDS at position 4544 were found in the *MUC6* fragment from tumour sample ID 47. (C) The absence of mutation A>G in CDS at position 4525 and the presence of a mutation C>A in CDS at position 4544 were found in the *MUC6* fragment from margin tissue sample ID 48. (D) The presence of mutation A>G in CDS at position 4525 and presence of mutation C>A in CDS at position 4544 were found in the *MUC6* fragment from margin tissue sample ID 90. (E) The presence of mutation T>A in CDS at position 12272 was found in the *MUC16* fragment from tumour sample ID 37. (F) The presence of mutation T>A in CDS at position 12272 was found in the *MUC16* fragment from tumour sample ID 47. (G) The presence of mutation T>A in CDS at position 12272 was found in the *MUC16* fragment from margin tissue sample ID 48. (H) The presence of mutation T>A in CDS at position 12272 was found in the *MUC16* fragment from margin tissue sample ID 90. Electropherograms of Sanger sequencing traces were prepared in GeneStudio™ Pro 2.2.0.0 software (GeneStudio Inc., Suwanee, GA, USA).

Table S1. Accepted and rejected sequencing results.

	Tumour samples			Margin samples		
**Patient**	**ID**	***MUC6* sequencing**	***MUC16* sequencing**	**ID**	***MUC6* sequencing**	***MUC16* sequencing**
1	11	**Yes**	**Yes**	12	No	No
2	15	No	**Yes**	16	**Yes**	**Yes**
3	25	No	No	26	**Yes**	No
4	29	**Yes**	No	30	No	**Yes**
5	33	No	**Yes**	34	**Yes**	No
6	37	**Yes**	**Yes**	38	No	No
7	39	**Yes**	No	40	No	No
8	41	No	**Yes**	42	**Yes**	**Yes**
9	45	**Yes**	No	46	No	No
10	47	**Yes**	**Yes**	48	**Yes**	**Yes**
11	49	**Yes**	**Yes**	50	**Yes**	**Yes**
12	53	**Yes**	No	54	**Yes**	**Yes**
13	55	**Yes**	No	56	**Yes**	**Yes**
14	57	**Yes**	**Yes**	58	**Yes**	**Yes**
15	61	**Yes**	No	62	**Yes**	**Yes**
16	63	No	**Yes**	64	**Yes**	**Yes**
17	79	**Yes**	**Yes**	80	**Yes**	**Yes**
18	89	No	No	90	**Yes**	**Yes**

ID - sample identifier

Table S2. Detailed information of investigated *MUC6* (ENSG00000184956) mutations.

SAMPLE ID	SAMPLE TYPE	CHROM	POS	REF	ALT	HGVSc	HGVSp	Consequence	IMPACT	Amino acids	Existing variation	VARIANT CLASS	SIFT	PolyPhen
**11**	G	chr11	1018257	A	T	ENST00000421673.7:c.4544C>A	ENSP00000406861.2:p.Leu1515Ter	stop gained	HIGH	L/*		SNV		
**11**	G	chr11	1018258	C	T	ENST00000421673.7:c.4543T>A	ENSP00000406861.2:p.Ala1515Thr	missense variant	MODERATE	A/T		SNV	deleterious low confidence (0.04)	benign (0.026)
**16**	M	chr11	1018257	A	T	ENST00000421673.7:c.4544C>A	ENSP00000406861.2:p.Leu1515Ter	stop gained	HIGH	L/*		SNV		
**29**	G	chr11	1018257	A	T	ENST00000421673.7:c.4544C>A	ENSP00000406861.2:p.Leu1515Ter	stop gained	HIGH	L/*		SNV		
**34**	M	chr11	1018257	A	T	ENST00000421673.7:c.4544C>A	ENSP00000406861.2:p.Leu1515Ter	stop gained	HIGH	L/*		SNV		
**34**	M	chr11	1018465	T	C	ENST00000421673.7:c.4336C>G	ENSP00000406861.2:p.Asn1446Asp	missense variant	MODERATE	N/D	COSV70139063	SNV	deleterious (0.02)	benign (0.019)
**39**	G	chr11	1018257	A	T	ENST00000421673.7:c.4544C>A	ENSP00000406861.2:p.Leu1515Ter	stop gained	HIGH	L/*		SNV		
**39**	G	chr11	1018322	A	G	ENST00000421673.7:c.4479A>C	ENSP00000406861.2:p.Thr1493%3D	synonymous variant	LOW	T		SNV		
**39**	G	chr11	1018506	C	T,A	ENST00000421673.7:c.4295C>A	ENSP00000406861.2:p.Ser1432Asn	missense variant	MODERATE	S/N		SNV	tolerated low confidence (0.14)	benign (0.212)
**42**	M	chr11	1018257	A	T	ENST00000421673.7:c.4544C>A	ENSP00000406861.2:p.Leu1515Ter	stop gained	HIGH	L/*		SNV		
**42**	M	chr11	1018543	A	G	ENST00000421673.7:c.4258G>C	ENSP00000406861.2:p.Cys1420Arg	missense variant	MODERATE	C/R		SNV	tolerated (0.06)	probably damaging (0.921)
**45**	G	chr11	1018111	C	T,A	ENST00000421673.7:c.4690G>A	ENSP00000406861.2:p.Ala1564Thr	missense variant	MODERATE	A/T		SNV	tolerated (0.2)	benign (0.007)
**45**	G	chr11	1018257	A	T	ENST00000421673.7:c.4544C>A	ENSP00000406861.2:p.Leu1515Ter	stop gained	HIGH	L/*		SNV		
**47**	G	chr11	1018243	C	T		ENSP00000406861.2:p.Glu1520Lys	missense variant	MODERATE	E/K		SNV		
**47**	G	chr11	1018257	A	T	ENST00000421673.7:c.4544C>A	ENSP00000406861.2:p.Leu1515Ter	stop gained	HIGH	L/*		SNV		
**48**	M	chr11	1018257	A	T	ENST00000421673.7:c.4544C>A	ENSP00000406861.2:p.Leu1515Ter	stop gained	HIGH	L/*		SNV		
**50**	M	chr11	1018257	A	T	ENST00000421673.7:c.4544C>A	ENSP00000406861.2:p.Leu1515Ter	stop gained	HIGH	L/*		SNV		
**53**	G	chr11	1018257	A	T	ENST00000421673.7:c.4544C>A	ENSP00000406861.2:p.Leu1515Ter	stop gained	HIGH	L/*		SNV		
**54**	M	chr11	1018257	A	T	ENST00000421673.7:c.4544C>A	ENSP00000406861.2:p.Leu1515Ter	stop gained	HIGH	L/*		SNV		

SAMPLE ID	SAMPLE TYPE	CHROM	POS	REF	ALT	HGVSc	HGVSp	Consequence	IMPACT	Amino acids	Existing variation	VARIANT CLASS	SIFT	PolyPhen
**55**	G	chr11	1018180	C	T	ENST00000421673.7:c. 4621T>A	ENSP00000406861.2:p. Ala1541Thr	missense variant	MODERATE	A/T		SNV	tolerated (0.86)	benign (0.021)
**55**	G	chr11	1018257	A	T	ENST00000421673.7:c. 4544C>A	ENSP00000406861.2:p. Leu1515Ter	stop gained	HIGH	L/*		SNV		
**56**	M	chr11	1018381	C	T	ENST00000421673.7:c. 4420G>A	ENSP00000406861.2:p. Ala1474Thr	missense variant	MODERATE	A/T		SNV	tolerated low confidence (0.6)	benign (0)
**56**	M	chr11	1018513	C	A,T	ENST00000421673.7:c. 4288A>T	ENSP00000406861.2:p. Ala1430Ser	missense variant	MODERATE	A/S		SNV	tolerated low confidence (0.21)	benign (0.045)
**57**	G	chr11	1018257	A	T	ENST00000421673.7:c. 4544C>A	ENSP00000406861.2:p. Leu1515Ter	stop gained	HIGH	L/*		SNV		
**58**	M	chr11	1018257	A	T	ENST00000421673.7:c. 4544C>A	ENSP00000406861.2:p. Leu1515Ter	stop gained	HIGH	L/*		SNV		
**58**	M	chr11	1018297	G	A	ENST00000421673.7:c. 4504A>T	ENSP00000406861.2:p. Leu1502%3D	synonymous variant	LOW	L	COSV70138702	SNV		
**61**	G	chr11	1018257	A	T	ENST00000421673.7:c. 4544C>A	ENSP00000406861.2:p. Leu1515Ter	stop gained	HIGH	L/*		SNV		
**62**	M	chr11	1018257	A	T	ENST00000421673.7:c. 4544C>A	ENSP00000406861.2:p. Leu1515Ter	stop gained	MODERATE	L/*		SNV		
**64**	M	chr11	1018257	A	T	ENST00000421673.7:c. 4544C>A	ENSP00000406861.2:p. Leu1515Ter	stop gained	HIGH	L/*		SNV		
**79**	G	chr11	1018257	A	T	ENST00000421673.7:c. 4544C>A	ENSP00000406861.2:p. Leu1515Ter	stop gained	HIGH	L/*		SNV		
**90**	M	chr11	1018257	A	T	ENST00000421673.7:c. 4544C>A	ENSP00000406861.2:p. Leu1515Ter	stop gained	HIGH	L/*		SNV		
**90**	M	chr11	1018276	T	C, A	ENST00000421673.7:c. 4525A>G	ENSP00000406861.2:p. Thr1509Ala	missense variant	HIGH	T/A		SNV	deleterious low confidence (0.02)	benign (0.037)

SAMPLE ID - sample identifier; **CHROM** - chromosome number; **POS -** start coordinate of the variant; **REF** - reference allele; **ALT** - alternative allele; **Consequence** - probable effect of this variant; **IMPACT** - classification of the severity of this variant consequence; **Amino acids** - the change in amino acids in the protein coding sequence (reference/alteration); ; **Existing variation** - identifier available in database (dbSNP or COSMIC); **Variant class** Sequence Ontology variant class (single nucleotide variation – SNV, insertion, or deletion). **SIFT** - Scale Invariant Feature Transform prediction and score; **PolyPhen** - Polymorphism Phenotyping v2 prediction and score

Table S3. Detailed information of investigated *MUC16* (ENSG00000181143) mutations.

SAMPLE ID	SAMPLE TYPE	CHROM	POS	REF	ALT	HGVSc	HGVSp	Consequence	IMPACT	Amino acids	Existing variation	VARIANT CLASS	SIFT	PolyPhen
**11**	G	chr19	8964498	A	T	ENST00000397910.8:c.12272T>A	ENSP00000381008.2:p.Met4091Lys	missense variant	MODERATE	M/K		SNV	deleterious low confidence (0)	benign (0.028)
**11**	G	chr19	8964755	A	C,T	ENST00000397910.8:c.12015T>G	ENSP00000381008.2:p.Ala4005%3D	synonymous variant	LOW	A		SNV		
**15**	G	chr19	8964498	A	T	ENST00000397910.8:c.12272T>A	ENSP00000381008.2:p.Met4091Lys	missense variant	MODERATE	M/K		SNV	deleterious low confidence (0)	benign (0.028)
**15**	G	chr19	8964627	A	T	ENST00000397910.8:c.12143G>A	ENSP00000381008.2:p.Ile4048Lys	missense variant	MODERATE	I/K		SNV	tolerated low confidence (0.09)	benign (0.013)
**16**	M	chr19	8964498	A	T	ENST00000397910.8:c.12272T>A	ENSP00000381008.2:p.Met4091Lys	missense variant	MODERATE	M/K		SNV	deleterious low confidence (0)	benign (0.028)
**30**	M	chr19	8964498	A	T	ENST00000397910.8:c.12272T>A	ENSP00000381008.2:p.Met4091Lys	missense variant	MODERATE	M/K		SNV	deleterious low confidence (0)	benign (0.028)
**33**	G	chr19	8964498	A	T	ENST00000397910.8:c.12272T>A	ENSP00000381008.2:p.Met4091Lys	missense variant	MODERATE	M/K		SNV	deleterious low confidence (0)	benign (0.028)
**33**	G	chr19	8964627	A	T	ENST00000397910.8:c.12143G>A	ENSP00000381008.2:p.Ile4048Lys	missense variant	MODERATE	I/K		SNV	tolerated low confidence (0.09)	benign (0.013)
**37**	G	chr19	8964498	A	T	ENST00000397910.8:c.12272T>A	ENSP00000381008.2:p.Met4091Lys	missense variant	MODERATE	M/K		SNV	deleterious low confidence (0)	benign (0.028)
**37**	G	chr19	8964627	A	T	ENST00000397910.8:c.12143G>A	ENSP00000381008.2:p.Ile4048Lys	missense variant	MODERATE	I/K		SNV	tolerated low confidence (0.09)	benign (0.013)
**47**	G	chr19	8964498	A	T	ENST00000397910.8:c.12272T>A	ENSP00000381008.2:p.Met4091Lys	missense variant	MODERATE	M/K		SNV	deleterious low confidence (0)	benign (0.028)
**48**	M	chr19	8964498	A	T	ENST00000397910.8:c.12272T>A	ENSP00000381008.2:p.Met4091Lys	missense variant	MODERATE	M/K		SNV	deleterious low confidence (0)	benign (0.028)
**56**	M	chr19	8964498	A	T	ENST00000397910.8:c.12272T>A	ENSP00000381008.2:p.Met4091Lys	missense variant	MODERATE	M/K		SNV	deleterious low confidence (0)	benign (0.028)
**57**	G	chr19	8964498	A	T	ENST00000397910.8:c.12272T>A	ENSP00000381008.2:p.Met4091Lys	missense variant	MODERATE	M/K		SNV	deleterious low confidence (0)	benign (0.028)
**62**	M	chr19	8964498	A	T	ENST00000397910.8:c.12272T>A	ENSP00000381008.2:p.Met4091Lys	missense variant	MODERATE	M/K		SNV	deleterious low confidence (0)	benign (0.028)
**63**	G	chr19	8964498	A	T	ENST00000397910.8:c.12272T>A	ENSP00000381008.2:p.Met4091Lys	missense variant	MODERATE	M/K		SNV	deleterious low confidence (0)	benign (0.028)
**63**	G	chr19	8964627	A	T	ENST00000397910.8:c.12143G>A	ENSP00000381008.2:p.Ile4048Lys	missense variant	MODERATE	I/K		SNV	tolerated low confidence (0.09)	benign (0.013)
**64**	M	chr19	8964498	A	T	ENST00000397910.8:c.12272T>A	ENSP00000381008.2:p.Met4091Lys	missense variant	MODERATE	M/K		SNV	deleterious low confidence (0)	benign (0.028)
**64**	M	chr19	8964627	A	T	ENST00000397910.8:c.12143G>A	ENSP00000381008.2:p.Ile4048Lys	missense variant	MODERATE	I/K		SNV	tolerated low confidence (0.09)	benign (0.013)

SAMPLE ID	SAMPLE TYPE	CHROM	POS	REF	ALT	HGVSc	HGVSp	Consequence	IMPACT	Amino acids	Existing variation	VARIANT CLASS	SIFT	PolyPhen
**80**	M	chr19	8964498	A	T	ENST00000397910.8:c.12272T>A	ENSP00000381008.2:p.Met4091Lys	missense variant	MODERATE	M/K		SNV	deleterious low confidence (0)	benign (0.028)
**90**	M	chr19	8964498	A	T	ENST00000397910.8:c.12272T>A	ENSP00000381008.2:p.Met4091Lys	missense variant	MODERATE	M/K		SNV	deleterious low confidence (0)	benign (0.028)

SAMPLE ID - sample identifier; **CHROM** - chromosome number; **POS -** start coordinate of the variant; **REF** - reference allele; **ALT** - alternative allele; **Consequence** - probable effect of this variant; **IMPACT** - classification of the severity of this variant consequence; **Amino acids** - the change in amino acids in the protein coding sequence (reference/alteration); ; **Existing variation** - identifier available in database (dbSNP or COSMIC); **Variant class** Sequence Ontology variant class (single nucleotide variation – SNV, insertion, or deletion). **SIFT** - Scale Invariant Feature Transform prediction and score; **PolyPhen** - Polymorphism Phenotyping v2 prediction and score
